# The burden of health conditions for middle-aged and older adults in the United States: disability-adjusted life years

**DOI:** 10.1186/s12877-019-1110-6

**Published:** 2019-04-08

**Authors:** Ryan McGrath, Soham Al Snih, Kyriakos Markides, Orman Hall, Mark Peterson

**Affiliations:** 1 Department of Health, Nutrition, and Exercise Sciences, North Dakota State University, Fargo, ND USA; 2 Division of Rehabilitation Sciences, University of Texas Medical Branch, Galveston, TX USA; 3 Department of Preventive Medicine and Community Health, University of Texas Medical Branch, Galveston, TX USA; 4 Department of Physical Medicine and Rehabilitation, University of Michigan, Ann Arbor, MI USA

**Keywords:** Normative aging, Epidemiology, Longevity, Morbidity

## Abstract

**Background:**

Many adults are living longer with health conditions in the United States. Understanding the disability-adjusted life years (DALYs) for such health conditions may help to inform healthcare providers and their patients, guide health interventions, reduce healthcare costs, improve quality of life, and increase longevity for aging Americans. The purpose of this study was to determine the burden of 10 health conditions for a nationally-representative sample of adults aged 50 years and older in the United States.

**Methods:**

Data from the 1998–2014 waves of the Health and Retirement Study were analyzed. At each wave, participants indicated if they were diagnosed with the following 10 conditions: cancer, chronic obstructive pulmonary disease (COPD), congestive heart failure, diabetes, back pain, hypertension, a fractured hip, myocardial infarction, rheumatism or arthritis, and a stroke. Years lived with a disability and years of life lost to premature mortality were summed for calculating DALYs. Sample weights were utilized in the analyses to make the DALY estimates nationally-representative. Results for the DALYs were presented in thousands.

**Results:**

There were 30,101 participants included. Sex stratified DALY estimates ranged from 4092 (fractured hip)-to-178,055 (hypertension) for men and 13,621 (fractured hip)-to-200,794 (hypertension) for women. The weighted overall DALYs were: 17,660 for hip fractures, 62,630 for congestive heart failure, 64,710 for myocardial infarction, 90,337 for COPD, 93,996 for stroke, 142,012 for cancer, 117,534 for diabetes, 186,586 for back pain, 333,420 for arthritis, and 378,849 for hypertension. In total, there were an estimated 1,487,734 years of healthy life lost from the 10 health conditions examined over the study period.

**Conclusions:**

The burden of these health conditions accounted for over a million years of healthy life lost for middle-aged and older Americans over the 16 year study period. Our results should be used to inform healthcare providers and guide health interventions aiming to improve the health of middle-aged and older adults. Moreover, shifting health policy and resources to match DALY trends may help to improve quality of life during aging and longevity.

**Electronic supplementary material:**

The online version of this article (10.1186/s12877-019-1110-6) contains supplementary material, which is available to authorized users.

## Background

Increased age is a hallmark risk factor for several health conditions [1]. Although approximately 86% of older adults in the United States are living with at least one health condition [2], life expectancy in the United States has generally continued to increase [3]. The advancements in life expectancy have been attributed to many factors including improvements in the prevention and treatment of morbidity. Given that the older adult population is projected to grow 112% by the year 2060 [4], healthcare providers and policy makers need to continue accommodating the emerging health demands of this population for helping them live longer, and with more quality years. For example, public health programs have been developed for improving the wellbeing and longevity of aging adults. Healthy People 2020 and 2030 includes initiatives to prevent morbidity, improve quality of care, and delay mortality for older adults in the United States [5].

Disability-adjusted life years (DALYs) are used globally to quantify the number of healthy years of life lost from the presence of a disease, disability, or injury [6]. The burden of chronic, non-fatal health loss and early mortality is evaluated separately and compared across populations. Information for DALYs in the United States and globally is often provided in Global Burden of Disease studies [7]. Such information is used to inform healthcare providers about the impact of a health condition and guide interventions seeking to improve the health and life expectancy of a given population [8]. Being that this time-based metric measures the burden of a health condition in a population and compares to a healthy population that reaches full life expectancy, the specific burden of common health conditions for middle-aged and older adults in the United States has yet to be calculated.

More studies are needed for understanding how aging is linked with disease [1]. Calculating the years lived with a disease (YLDs) and years of life lost (YLLs) from premature mortality will provide insights into the burden of common health conditions for the growing aging adult population. This information can help to identify which health conditions contribute most to the number of healthy years of life lost for aging adults, thereby informing how healthcare providers and interventions prioritize treatment and prevention efforts. Such prioritization will help to guide health policy, and increase the quality of life and longevity for aging adults. Therefore, the purpose of this study was to determine the burden of 10 common health conditions for a nationally-representative sample of middle-aged and older adults in the United States.

## Methods

### Participants

Data from 37,495 participants in the 1998–2014 waves of the Health and Retirement Study (HRS) were used. Individual data files were joined to the cleaned and standardized RAND HRS dataset. The purpose of the HRS is to understand the health and economic implications of advancing age that can threaten or promote health and wealth at individual- and population-levels [9]. Participants in the HRS have been re-interviewed biennially since 1992. Further, the HRS includes surveys from over 23,000 households and has provided data for a nationally-representative sample of Americans aged over 50 years since 1998 [10]. New cohorts of participants have been added to the original HRS sample to preserve national representation and participants are followed longitudinally until death [10]. A multi-stage probability design is used by the HRS, including geographical stratification and oversampling of certain demographic groups. Additional details for the HRS are described elsewhere [11].

Written informed consent was acquired from all participants before entering the study and protocols were approved by the University of Michigan Behavioral Sciences Committee Institutional Review Board. Participant anonymity was ensured because data used in this secondary analysis contained no direct identifiers.

### Health conditions

Participants self-reported their date of birth and sex. Interviewers asked participants questions related to their physical health at each wave. Individuals who reported having cancer, chronic obstructive pulmonary disease (COPD), congestive heart failure, diabetes or high blood sugar, back pain, high blood pressure or hypertension, a fractured hip, a heart attack or myocardial infarction, rheumatism or arthritis, or a stroke were included. The date of interview for the wave a health condition was first confirmed by participants was treated as a proxy for the date of diagnosis. These health conditions were selected by investigators because they were identified as having a corresponding disability weight from the Global Burden of Disease [12, 13]. Example interviewer questions for each health condition that were asked to participants are listed in Additional file 1: Table S1.

### Mortality

Date of death was obtained through linkage to the National Death Index. The HRS also conducted an interview with a surviving spouse, child, or other informant for each decedent, successfully obtaining study exit information [10].

### Statistical analysis

Procedures from the World Health Organization for determining DALYs with an incidence-based calculation were used [14]. Participants were first stratified by sex, then by age categories (50–59 years, 60–69 years, 70–79 years, ≥80 years). The age at which a health condition occurred determined age categories for all participants.

YLDs were calculated by multiplying the number of incident cases for each health condition, corresponding disability weight, and average duration of years lived with the health condition until death, or truncation. For those who were still alive or lost to follow-up (i.e., truncation), the average duration of years lived with the health condition was determined using their estimated life expectancy at age of truncation [15]. Disability weights for each health condition were from the Global Burden of Disease (back pain = 0.020, cancer = 0.288, COPD = 0.019, congestive heart failure = 0.201, diabetes = 0.015, fractured hip = 0.058, hypertension = 0.246, myocardial infarction = 0.439, rheumatism or arthritis =0.199, stroke = 0.266) [12, 13]. For each sex, YLDs were summed across age categories to determine total YLDs.

YLLs were calculated by taking the product of the number of deaths that occurred by the mean life expectancy at age of death in years. The Period Life Table was used to determine life expectancy at each age for men and women [16]. The YLLs were summed across age categories to determine total YLLs.

For men and women, YLDs and YLLs were added across age categories to determine DALYs for each health condition. Then, the DALYs estimates were summed for calculating overall DALYs. Sample weights were utilized in the analyses so DALYs were nationally-representative. The YLLs, YLDs, and DALYs are reported in thousands. All analyses were performed with SAS 9.4 software (SAS Institute; Cary, NC).

## Results

**Fig. 1:**
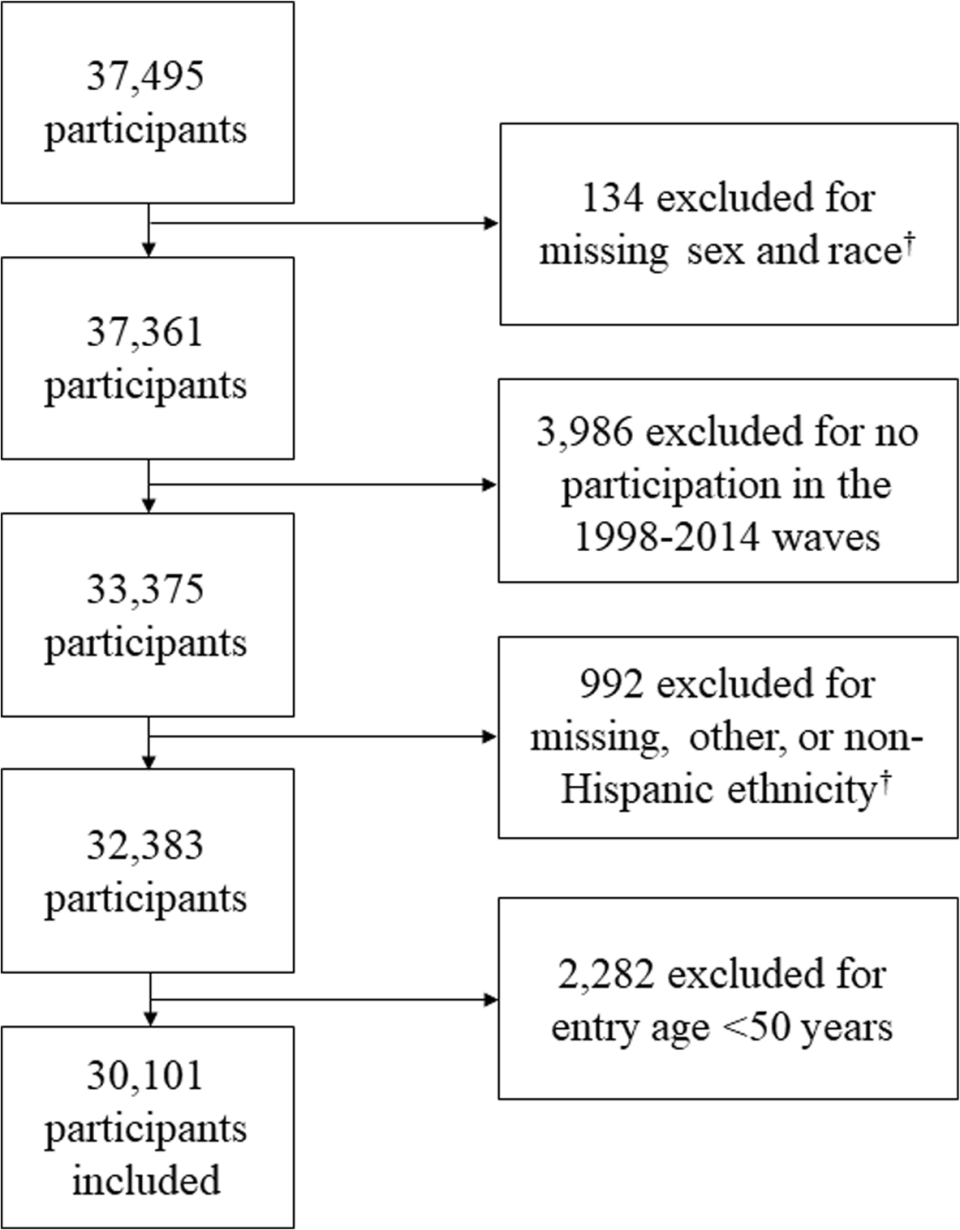
Flow Chart for Exclusions. ^†^Exclusions occurred because races and ethnicities in the other category were stratified

After exclusions (Fig. 1), there were 30,101 participants included (*n* = 16,591 women, *n* = 13,510 men) from the 1998–2014 waves who reported having at least one of the health conditions we examined. The non-weighted and weighted descriptive characteristics of the participants are presented in Table 1. Overall, participants entered the study at 63.3 ± 10.6 years of age. Of the 10,504 participants that died, the age at death was 79.6 ± 10.5 years. Table 2 provides person-level DALY estimates and 95% confidence intervals for each health outcome.

**Table 1:** Non-Weighted and Weighted Descriptive Characteristics of the Participants

	Overall (*n* = 30,101)	Weighted Overall (*n* = 114,610,740)	Women (*n* = 16,591)	Weighted Women (*n* = 59,183,770)	Men (*n* = 13,510)	Weighted Men (*n* = 55,426,970)
Age (years)	63.3 ± 10.6	60.9 ± 10.0	63.7 ± 11.1	61.8 ± 10.6	62.9 ± 10.0	60.1 ± 9.4
Age at Death (years)	79.6 ± 10.5	78.4 ± 11.0	81.2 ± 10.7	80.4 ± 10.8	77.9 ± 10.1	76.3 ± 10.9
Died (n (%))	10,504 (34.9%)	31,954,223 (27.8%)	5553 (52.8%)	16,504,100 (27.9%)	4951 (16.4%)	15,450,123 (27.8%)

**Table 2:** Person-Level Disability-Adjusted Life Year Means and 95% Confidence Intervals

	Mean	95% Confidence Interval	Mean	95% Confidence Interval	Mean	95% Confidence Interval
	Arthritis		Back Pain		Cancer	
Males						
50–59 Years	20.4	20.1, 20.8	20.6	20.2, 21.0	21.9	21.4, 22.5
60–69 Years	14.4	14.2, 14.6	13.4	13.2, 13.7	15.7	15.4, 16.0
70–79 Years	9.1	9.0, 9.2	8.4	8.2, 8.6	10.1	10.0, 10.3
≥80 Years	5.4	5.3, 5.4	4.5	4.4, 4.7	5.8	5.7, 6.0
Total	11.0	10.9, 11.2	10.5	10.2, 10.7	11.2	10.9, 11.4
Females						
50–59 Years	23.0	22.7, 23.4	23.0	22.5, 23.5	24.8	24.2, 25.3
60–69 Years	16.1	15.9, 16.3	15.1	14.8, 15.4	17.5	17.2, 17.8
70–79 Years	9.9	9.8, 10.0	9.2	9.0, 9.4	11.1	10.9, 11.3
≥80 Years	5.8	5.7, 5.9	5.0	4.8, 5.1	6.5	6.4, 6.6
Total	11.0	10.8, 11.2	10.5	10.3, 10.7	12.5	12.2, 12.8
Overall						
50–59 Years	21.8	21.6, 22.1	21.8	21.4, 22.1	23.5	23.1, 24.0
60–69 Years	15.2	15.1, 15.4	14.3	14.1, 14.5	16.6	16.4, 16.8
70–79 Years	9.5	9.5, 9.6	8.8	8.7, 9.0	10.6	10.5, 10.7
≥80 Years	5.7	5.6, 5.7	4.8	4.7, 4.9	6.2	6.1, 6.3
Total	11.0	10.9, 11.2	10.5	10.3, 10.7	11.8	11.6, 12.1
	Chronic Obstructive Pulmonary Disease		Congestive Heart Failure		Diabetes	
Males						
50–59 Years	19.8	19.1, 20.4	21.7	20.9, 22.4	19.6	19.1, 20.1
60–69 Years	13.5	13.2, 13.9	15.4	15.0, 15.8	13.4	13.1, 13.7
70–79 Years	8.6	8.4, 8.8	9.8	9.6, 10.0	8.2	8.0, 8.4
≥80 Years	5.0	4.8, 5.1	5.2	5.1, 5.4	4.8	4.6, 5.0
Total	10.4	10.1, 10.7	10.4	10.0, 10.8	11.1	10.8, 11.3
Females						
50–59 Years	21.9	21.2, 22.6	24.5	23.7, 25.3	22.0	21.5, 22.6
60–69 Years	15.6	15.2, 16.0	17.2	16.7, 17.7	15.0	14.7, 15.3
70–79 Years	9.6	9.3, 9.8	11.0	10.7, 11.3	9.1	8.9, 9.3
≥80 Years	5.3	5.1, 5.5	5.8	5.6, 5.9	5.3	5.1, 5.4
Total	11.5	11.2, 11.9	10.4	10.2, 10.7	11.9	11.6, 12.2
Overall						
50–59 Years	20.9	20.4, 21.4	23.1	22.5, 23.7	20.8	20.4, 21.2
60–69 Years	14.6	14.3, 14.8	16.3	16.0, 16.6	14.2	14.0, 14.4
70–79 Years	9.1	8.9, 9.2	10.4	10.2, 10.6	8.6	8.5, 8.8
≥80 Years	5.1	5.0, 5.3	5.6	5.5, 5.7	5.1	4.9, 5.2
Total	11.0	10.7, 11.2	10.4	10.1, 10.6	11.5	11.3, 11.7
	Fractured Hip		Hypertension		Myocardial Infarction	
Males						
50–59 Years	–	–	21.3	21.0, 21.6	22.4	21.8, 22.9
60–69 Years	13.6	12.6, 14.5	14.8	14.6, 15.0	16.5	16.2, 16.8
70–79 Years	9.0	8.4, 9.5	9.5	9.4, 9.6	10.9	10.7, 11.1
≥80 Years	4.8	4.5, 5.1	5.7	5.6, 5.8	6.5	6.4, 6.7
Total	7.2	6.7, 7.7	11.9	11.8, 12.1	11.9	11.5, 12.2
Females						
50–59 Years	–	–	23.8	23.5, 24.1	25.0	24.1, 25.8
60–69 Years	15.7	14.8, 16.6	16.6	16.4, 16.8	18.2	17.8, 18.7
70–79 Years	10.1	9.6, 10.5	10.3	10.2, 10.4	12.0	11.8, 12.3
≥80 Years	5.0	4.8, 5.2	6.1	6.0, 6.2	6.9	6.7, 7.1
Total	6.8	6.5, 7.1	11.7	11.5, 11.9	11.8	11.4, 12.3
Overall						
50–59 Years	–	–	22.5	22.3, 22.7	23.5	22.9, 24.0
60–69 Years	15.0	14.3, 15.7	15.7	15.6, 15.8	17.1	16.9, 17.4
70–79 Years	9.7	9.3, 10.0	9.9	9.8, 10.0	11.4	11.2, 11.6
≥80 Years	5.0	4.8, 5.1	5.9	5.9, 6.0	6.7	6.6, 6.8
Total	6.9	6.7, 7.2	11.8	11.7, 12.0	11.8	11.6, 12.1
	Stroke					
Males						
50–59 Years	21.5	20.8, 22.1				
60–69 Years	15.8	15.4, 16.1				
70–79 Years	9.7	9.5, 9.8				
≥80 Years	5.5	5.3, 5.6				
Total	10.6	10.3, 10.9				
Females						
50–59 Years	24.4	23.7, 25.1				
60–69 Years	17.4	17.0, 17.8				
70–79 Years	11.0	10.8, 11.2				
≥80 Years	6.1	6.0, 6.2				
Total	10.1	9.8, 10.4				
Overall						
50–59 Years	22.9	22.3, 23.4				
60–69 Years	16.5	16.2, 16.8				
70–79 Years	10.3	10.2, 10.5				
≥80 Years	5.9	5.8, 6.0				
Total	10.3	10.1, 10.5				

**Fig. 2:**
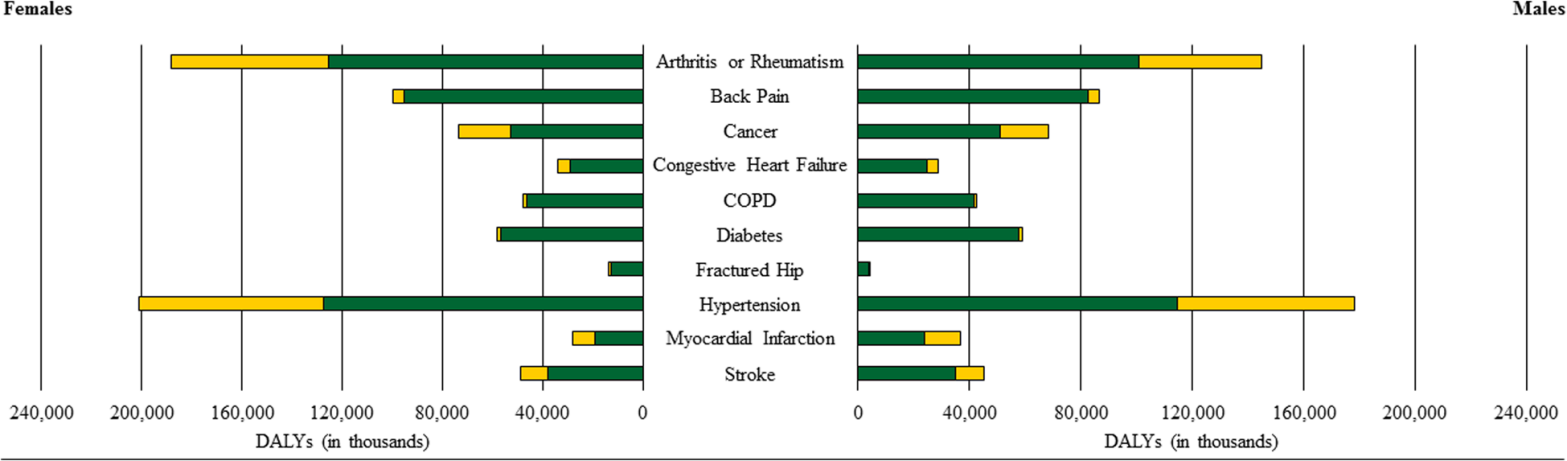
The Burden of the Health Outcomes for Middle-Aged and Older Adults Stratified by Sex. Note: Green Bars = Years of Life Lost; Yellow Bars = Years Lived with Disease. COPD = Chronic Obstructive Pulmonary Disease; DALYs = Disability-Adjusted Life Years

Figure 2 presents the weighted YLDs and YLLs for each health condition stratified by sex. DALY estimates for men were: 4092 for hip fractures, 28,707 for congestive heart failure, 36,688 for myocardial infarction, 42,413 for COPD, 45,197 for stroke, 59,006 for diabetes, 68,237 for cancer, 86,392 for back pain, 144,991 for arthritis, and 178,055 for hypertension. Likewise, DALY estimates for women were: 13,621 for hip fractures, 27,855 for myocardial infarction, 33,874 for congestive heart failure, 47,802 for COPD, 48,587 for stroke, 58,101 for diabetes, 73,529 for cancer, 99,736 for back pain, 188,177 for arthritis, and 200,794 for hypertension. Of the ten health conditions examined herein, the number of DALYs for diabetes and myocardial infarction were only higher in men than women. In total, the 10 health conditions accounted for an estimated 693,778 DALYs in men and 792,076 DALYs in women.

The weighted overall DALYs were: 17,660 for hip fractures, 62,630 for congestive heart failure, 64,710 for myocardial infarction, 90,337 for COPD, 93,996 for stroke, 117,534 for diabetes, 142,012 for cancer, 186,586 for back pain, 333,420 for arthritis, and 378,849 for hypertension. Detailed information for the weighted burden of each health condition by sex and overall is presented in Table 3. As a whole, there were an estimated 347,274 YLDs, 1,140,457 YLLs, and 1,487,734 DALYs for the 10 health conditions.

**Table 3:** Disability-Adjusted Life Years for Each Health Outcome

	Cases	Number Dead	YLLs (in thousands)	YLDs (in thousands)	DALYs (in thousands)
Arthritis					
Males					
50–59 Years	13,140,604	1,767,444	33,628	19,243	52,871
60–69 Years	9,293,034	2,465,646	31,798	14,254	46,053
70–79 Years	5,768,620	3,415,221	26,344	8051	34,396
≥80 Years	2,600,532	2,069,681	9260	2411	11,671
Total	30,802,790	9,717,992	101,030	43,959	144,991
Females					
50–59 Years	16,094,235	1,583,622	34,047	25,641	59,688
60–69 Years	11,064,877	2,422,174	35,011	18,417	53,428
70–79 Years	8,296,633	4,304,371	36,176	13,157	49,333
≥80 Years	5,291,347	4,199,135	20,000	5728	25,728
Total	40,747,092	12,509,302	125,234	62,943	188,177
Overall					
50–59 Years	29,234,839	3351,066	68,226	44,842	113,068
60–69 Years	20,357,911	4,887,820	66,848	32,557	99,405
70–79 Years	14,065,253	7,719,592	62,446	21,085	83,532
≥80 Years	7,891,879	6,268,816	29,270	8145	37,415
Total	71,549,882	22,227,294	226,790	106,629	333,420
Back Pain					
Males					
50–59 Years	15,686,707	1,740,053	35,669	1980	37,648
60–69 Years	6,832,356	1,653,968	22,088	932	23,021
70–79 Years	4,396,870	2,113,526	17,575	530	18,105
≥80 Years	2,184,198	1,645,791	7424	194	7618
Total	29,100,131	7,153,338	82,756	3636	86,392
Females					
50–59 Years	15,354,395	1,241,525	28,443	2126	30,569
60–69 Years	8,568,148	1,788,250	26,819	1221	28,040
70–79 Years	6,382,777	2,705,337	24,726	881	25,607
≥80 Years	4,351,801	3,076,734	15,084	436	15,520
Total	34,657,121	8,811,846	95,072	4664	99,736
Overall					
50–59 Years	31,041,102	2,981,578	64,644	4104	68,747
60–69 Years	15,400,504	3,442,218	48,969	2151	51,120
70–79 Years	10,779,647	4,818,863	42,198	1400	43,598
≥80 Years	6,535,999	4,722,525	22,493	628	23,121
Total	63,757,252	15,965,184	178,304	8283	186,586
Cancer					
Males					
50–59 Years	2,522,588	636,050	12,994	3863	16,857
60–69 Years	3,755,041	1,213,733	17,078	6250	23,328
70–79 Years	3,472,371	1,731,529	14,875	5443	20,318
≥80 Years	1,618,157	1,236,842	5880	1854	7734
Total	11,368,157	4,818,154	50,827	17,410	68,237
Females					
50–59 Years	3,700,463	591,564	13,619	6663	20,282
60–69 Years	3,429,015	1,027,035	16,203	6220	22,423
70–79 Years	3,072,482	1,655,490	15,535	5413	20,948
≥80 Years	1,988,047	1385,791	7186	2690	9876
Total	12,190,007	4,659,880	52,543	20,986	73,529
Overall					
50–59 Years	6,223,051	1,227,614	26,898	10,487	37,385
60–69 Years	7,184,056	2,240,768	33,349	12,469	45,818
70–79 Years	6544,853	3,387,019	30,372	10,862	41,234
≥80 Years	3,606,204	2,622,633	13,055	4520	17,575
Total	23,558,164	9,478,034	103,674	38,338	142,012
Chronic Obstructive Pulmonary Disease					
Males					
50–59 Years	2,580,113	632,447	12,445	316	12,762
60–69 Years	2,591,636	986,541	13,276	324	13,600
70–79 Years	2,108,494	1,366,288	11,661	208	11,869
≥80 Years	1,039,325	830,476	4116	67	4182
Total	8,319,568	3,815,752	41,498	915	42,413
Females					
50–59 Years	3,470,551	624,008	13,626	478	14,104
60–69 Years	2,847,547	906,558	14,095	375	14,469
70–79 Years	2,563,061	1,422,684	13,507	298	13,805
≥80 Years	1,407,161	1,011,150	5312	112	5424
Total	10,288,320	3,964,400	46,540	1263	47,802
Overall					
50–59 Years	6,050,664	1,256,455	26,209	792	27,001
60–69 Years	5,439,183	1,893,099	27,426	697	28,124
70–79 Years	4,671,555	2,788,972	25,102	501	25,604
≥80 Years	2,446,486	1,841,626	9430	178	9608
Total	18,607,888	7,780,152	88,167	2168	90,337
Congestive Heart Failure					
Males					
50–59 Years	901,813	309,556	6407	928	7335
60–69 Years	1,093,609	452,061	6563	1020	7584
70–79 Years	1,359,220	924,139	8239	1249	9487
≥80 Years	956,151	794,466	3742	559	4301
Total	4,310,793	2,480,222	24,951	3756	28,707
Females					
50–59 Years	783,103	218,141	5114	880	5994
60–69 Years	1,004,064	434,919	7028	1083	8111
70–79 Years	1,449,735	979,321	9791	1535	11,325
≥80 Years	1,716,177	1,411,609	7293	1151	8444
Total	4,953,079	3,043,990	29,226	4649	33,874
Overall					
50–59 Years	1,684,916	527,697	11,634	1813	13,447
60–69 Years	2,097,673	886,980	13,590	2107	15,697
70–79 Years	2,808,955	1,903,460	17,972	2772	20,744
≥80 Years	2,672,328	2,206,075	11,033	1709	12,742
Total	9,263,872	5,524,212	54,229	8401	62,630
Diabetes					
Males					
50–59 Years	6,357,757	1,136,872	22,201	652	22,853
60–69 Years	5,109,121	1,327,408	17,770	535	18,305
70–79 Years	3,110,939	1,702,148	13,938	291	14,229
≥80 Years	1,111,108	743,942	3546	73	3619
Total	15,688,925	4,910,370	57,455	1551	59,006
Females					
50–59 Years	5,545,897	856,217	18,801	635	19,436
60–69 Years	4,846,946	1,185,951	17,734	543	18,276
70–79 Years	3,287,663	1,603,639	14,515	340	14,855
≥80 Years	1,588,280	1,035,687	5430	104	5534
Total	15,268,786	4,681,494	56,480	1622	58,101
Overall					
50–59 Years	11,903,654	1,993,089	41,352	1292	42,644
60–69 Years	9,956,067	2,513,359	35,588	1078	36,666
70–79 Years	6,398,602	3,305,787	28,432	629	29,061
≥80 Years	2,699,388	1,779,629	8986	177	9163
Total	30,957,711	9,591,864	114,358	3176	117,534
Fractured Hip					
Males					
50–59 Years	0	0	–	–	–
60–69 Years	146,403	41,458	554	39	593
70–79 Years	380,325	226,413	1969	118	2087
≥80 Years	441,705	287,456	1343	70	1412
Total	968,433	555,327	3866	227	4092
Females					
50–59 Years	0	0	–	–	–
60–69 Years	324,722	115,798	1791	88	1879
70–79 Years	894,606	501,588	4931	267	5198
≥80 Years	1,814,079	1,272,571	6168	376	6544
Total	3,033,407	1,889,957	12,890	731	13,621
Overall					
50–59 Years	0	0	–	–	–
60–69 Years	471,125	157,256	2324	127	2451
70–79 Years	1,274,931	728,001	6868	386	7254
≥80 Years	2,255,784	1,560,027	7509	446	7955
Total	4,001,840	2,445,284	16,701	959	17,660
Hypertension					
Males					
50–59 Years	17,589,184	2,219,853	43,583	30,454	74,037
60–69 Years	10,044,721	2,652,896	34,384	18,925	53,310
70–79 Years	6,329,398	3,587,473	27,980	10,942	38,922
≥80 Years	2,551,064	1,933,743	8767	3018	11,786
Total	36,514,367	10,393,965	114,714	63,339	178,055
Females					
50–59 Years	15,159,062	1,560,757	34,321	28,232	62,554
60–69 Years	10,942,927	2,570,377	37,670	21,718	59,388
70–79 Years	8,588,794	4,388,589	36,871	16,804	53,674
≥80 Years	5,024,558	3,838,927	18,314	6864	25,178
Total	39,715,341	12,358,650	127,176	73,618	200,794
Overall					
50–59 Years	32,748,246	3,780,610	77,904	58,686	136,591
60–69 Years	20,987,648	5,223,273	72,054	40,643	112,698
70–79 Years	14,918,192	7,976,062	64,851	27,746	92,596
≥80 Years	7,575,622	5,772,670	27,081	9882	36,964
Total	76,229,708	22,752,615	241,890	136,957	378,849
Myocardial Infarction					
Males					
50–59 Years	1,477,509	311,855	6054	4451	10,505
60–69 Years	1,472,041	555,368	7866	3466	11,332
70–79 Years	1,322,100	781,906	6584	3304	9888
≥80 Years	844,074	721,969	3578	1385	4963
Total	5,115,724	2,371,098	24,082	12,606	36,688
Females					
50–59 Years	648,934	147,456	3181	2242	5422
60–69 Years	850,883	350,042	5548	2080	7628
70–79 Years	1,021,117	656,615	6217	2686	8903
≥80 Years	999,380	803,335	4090	1811	5902
Total	3,520,314	1,957,448	19,036	8819	27,855
Overall					
50–59 Years	2126,443	459,311	9333	6800	16,133
60–69 Years	2,322,924	905,410	13,392	5548	18,940
70–79 Years	2,343,217	1,438,521	12,783	5993	18,776
≥80 Years	1,843,454	1,525,304	7669	3193	10,861
Total	8,636,038	4,328,546	43,177	21,534	64,710
Stroke					
Males					
50–59 Years	1,566,132	450,841	8890	2850	11,739
60–69 Years	1,849,572	736,604	10,615	2581	13,195
70–79 Years	2,200,265	1,326,626	10,864	3351	14,215
≥80 Years	1,464,604	1,019,878	4668	1380	6048
Total	7,080,573	3,533,949	35,037	10,162	45,197
Females					
50–59 Years	1,257,018	250,971	5758	1891	7648
60–69 Years	1,644,126	581,634	9217	2658	11,875
70–79 Years	2,222,147	1,289,628	12,337	3357	15,694
≥80 Years	2,782,176	2,064,287	10,575	2794	13,370
Total	7,905,467	4,186,520	37,887	10,700	48,587
Overall					
50–59 Years	2,823,150	701,812	14,901	4718	19,619
60–69 Years	3,493,698	1,318,238	19,853	5224	25,077
70–79 Years	4,422,412	2,616,254	23,174	6709	29,883
≥80 Years	4,246,780	3,084,165	15,239	4178	19,417
Total	14,986,040	7,720,469	73,167	20,829	93,996

Note: *DALYs* Disability-Adjusted Life Years, *YLDs* Years Lived with Disease, *YLLs* Years of Life Lost

## Discussion

The principal findings of this investigation revealed that over 1-million years of healthy life were lost for middle-aged and older Americans from the 10 health conditions evaluated over the 16 year study period. Although aging adults were impacted by each health condition, hypertension accounted for the greatest burden; whereas, hip fractures had the lowest number of DALYs. These results were similar when evaluating the DALY estimates for each of the health conditions by sex. Our findings should be used to inform healthcare providers and interventions seeking to prevent morbidity and extend life expectancy in aging adults. Using DALYs to guide healthcare policy will also help to improve quality of life during aging through continued evolutions of disease prevention and treatment.

The Global Burden of Disease studies have identified hypertension as the leading risk factor by attributable disease burden [17]. The prevalence of hypertension increases with age, and is highest in older adults [18]. Of the ten health conditions evaluated in this investigation, hypertension had both the highest number of cases and DALYs. Likewise, those with hypertension had a large amount of YLDs, thereby indicating middle-aged and older adults are living with this disease for long periods of time after diagnosis. The large number of years lived with hypertension can be attributed to the evolution and adherence to hypertension medications [19, 20]. Like all medications, persons considering usage of promising hypertension medications should have discussions with a healthcare provider, and other non-pharmacological modes of treatment and prevention such as engaging in healthy behaviors remains a critical factor for reducing hypertension [18, 21]. Like hypertension, participants indicating they had arthritis or rheumatism also lived with this health condition for long periods of time after diagnosis as demonstrated by the large number of YLDs. These results align with another investigation that revealed rheumatoid arthritis causes significant YLDs and high overall disease burden [22]. It is projected that as smoking rates decline, the number of healthy years of life lost from rheumatoid arthritis will also decrease [22]. Future studies monitoring DALYs for arthritis in middle-aged and older adults are needed to confirm such projections and assess if arthritis medications lower the burden of arthritis in aging adults.

Back pain is generally a prevalent health condition all adults experience as they age and pain management is often challenging [23]. The health implications of back pain are also pronounced, as the Global Burden of Disease project demonstrated that back pain has a large burden in the United States, and is relatively lower in Asian countries [24, 25]. Although our results also suggest the burden of back pain is high for middle-aged and older adults in the United States, our findings for YLDs are lower compared to those of other similar investigations [24, 25]. We believe that this result is attributed to participants reporting back pain before entering the HRS, as indicated by the large number of cases for those aged 50–59 years. Cancer is also a leading cause of morbidity and mortality in older adults [26]. The rise of cancer rates for the older adult population in the United States is projected to increase, thereby posing challenges to healthcare systems and cancer patients [27]. Our results show that the burden of cancer in aging adults is high. Future investigations should continue monitoring DALYs for cancer and specific cancer types in aging adults to assess advancements in cancer treatment, care, and prevention.

About 33% of adults aged at least 65 years in the United States have diabetes and older adults with diabetes are at an elevated risk for mortality than those without diabetes [28]. According to the Global Burden of Disease, diabetes is a leading cause of DALYs in the United States [29], and men are more frequently diagnosed with diabetes than women at younger ages [30]. Our findings indicate the number of diabetes cases were higher in men than women, particularly at ages 50–59 and 60–69 years, which may explain why the burden of diabetes was higher for men than women. While our DALY estimates for diabetes were large, other countries in the Global Burden of Disease, such as Mexico, may have a higher burden from diabetes [25]. Similarly, our results revealed the number myocardial infarction cases and DALYs from this health condition were higher in men compared to women. These results align with another investigation that suggests the prevalence of myocardial infarctions is higher in men than women [31].

Stroke is a leading cause of disability and death for aging adults that is also responsible for billions of dollars in healthcare costs [32]. Persons that sustain a stroke have reduced mobility and are at an increased risk of experiencing another stroke [33]. Therefore, it is not unusual that the burden of stroke has remained high in the United States and globally [24, 25]. Our DALY results for stroke also indicate many healthy years of life lost in middle-aged and older adults. Although advancements in COPD prevention and treatment have been made [34], COPD remains a leading cause of death [35], and the Global Burden of Disease suggests COPD has a tremendous disease burden in the United States [29]. Given that COPD is progressive, persons living with this disease have a large amount of health-related costs [36]. While our findings indicate that the burden of COPD is already high, the burden of COPD is projected to increase [37]. As smoking cessation remains important for preventing and limiting the health effects of COPD, the burden of DALYs should continue to be monitored for helping to inform COPD treatments.

Heart failure is a worldwide health problem that is linked to high morbidity, mortality, and costs of care [38]. As the older adult population increases, the prevalence of heart diseases such as congestive heart failure has also risen [39]. Our results indicating the high burden of congestive heart failure are similar to those of other investigations evaluating DALYs [40]. Although hip fractures are common during aging, the incidence of hip fractures and mortality rates associated with hip fractures have declined in the United States [41, 42]. Another study evaluating DALYs for hip fractures determined that over 200,000 years of healthy life were lost from hip fractures in older adults [43]. While the burden of hip fractures was lowest of the ten health conditions for this investigation, prevention and treatment for hip fractures should remain a priority for aging adults.

Some limitations should be noted. Those who were lost from follow-up or died may have had a health condition that was not recorded before this event, thereby creating underestimations for our results. Moreover, the date of interview served as a proxy for diagnosis date, thereby allowing our results to be further underestimated. The use of an incidence-driven DALY calculation allowed us to determine how the burden of specific health conditions impacted middle-aged and older adults longitudinally; however, we were unable to control for multimorbiditiy in our disability weights. It is also possible that participants may have disputed their records for having a diagnosis or were no longer living with a health condition after initial diagnosis. Self-report biases may have occurred for participant responses. The HRS only includes adults aged 50 years and over; therefore, some participants may have had health conditions at younger ages before entering the study. Statistical tests of inference were not used for making comparisons between DALY estimates because DALYs are often used as a stand-alone statistic.

Likewise, our DALY estimates were influenced by cases, and YLD does not confirm that quality of life was compromised. Future investigations should examine the impact of a health condition on YLD because a smaller YLD may imply that a health condition exacerbates time to death; whereas, a larger YLD may suggest treatment and management of a health condition delays early mortality (depending on age of diagnosis and other important factors). As such, social and policy concerns for aging adults including living arrangements, finances, completion of autonomous living and basic self-care tasks, and care giving should be considered based on disease and health status. Comparing our findings with those of other burden of disease investigations performed in the United States and globally will be helpful for making comparisons across populations and diseases [44]. Moreover, expanding parts of the DALY calculation to other important health outcomes during aging and examining prevalence-based DALYs will help to advance our understanding of health burden.

## Conclusions

The burden of the health conditions evaluated for this investigation accounted for over a million years of healthy life lost for middle-aged and older Americans during the study period. Overall, participants experienced different levels of non-fatal health loss and early mortality for each health condition. These results should be used to help improve the efficiency and effectiveness of disease prevention and treatment strategies for aging adults. Trends in DALYs should continue to be monitored for middle-aged and older adults so that health-related policies and resources match DALY trends, and for informing healthcare providers so they can accommodate the health needs of the growing aging population in the United States. Encouraging healthcare providers to continue evolving prevention, treatment, and early detection for disease, and healthcare policy makers to invest in promising solutions will help to reduce health-related costs, improve quality of life, and extend life expectancy for the aging adults in the United States.

## Additional file

Additional file 1: **Table S1.** Example Interviewer Questions for Each Health Condition (DOCX 13 kb)

## Abbreviations

COPD: Chronic Obstructive Pulmonary Disease
DALY: Disability-Adjusted Life Year
HRS: Health and Retirement Study
YLD: Years Lived with Disease
YLL: Years of Life Lost
