# Supplementary material for: The burden of health conditions for middle-aged and older adults in the United States: disability-adjusted life years
Source: BMC Geriatr. 2019 Apr 8;19:100. doi: 10.1186/s12877-019-1110-6 (PMC6454610; doi:10.1186/s12877-019-1110-6)
Supplement: Supplementary file 1 — Table S1. Example Interviewer Questions for Each Health Condition (DOCX 13 kb) [file 12877_2019_1110_MOESM1_ESM.docx]

**Additional file 1: Table S1.** Example Interviewer Questions for Each Health Condition.

| **Health Condition** | **Question** |
| --- | --- |
| Back Pain | “Have you had any of the following persistent or troublesome problems? Back pain or problems?” |
| Hypertension | “Has a doctor ever told you that you have high blood pressure or hypertension?” |
| Cancer | “Has a doctor ever told you that you have cancer or a malignant tumor, excluding minor skin cancer?” |
| Chronic Obstructive Pulmonary Disease | “Has a doctor ever told you that you have chronic lung disease such as chronic bronchitis or emphysema (not including asthma)?” |
| Congestive Heart Failure | “Has a doctor told you that you have congestive heart failure?” |
| Diabetes or High Blood Sugar | “Has a doctor told you that you have diabetes or high blood sugar?” |
| Fractured Hip | “Have you fractured your hip since we talked?” |
| Myocardial Infarction | “Have you had a heart attack or myocardial infarction?” |
| Rheumatism or Arthritis | “Have you had or has a doctor told you that you have arthritis or rheumatism?” |
| Stroke | “Has a doctor told you that you have a stroke?” |
